# Editorial: Insights in motivation and reward - 2022

**DOI:** 10.3389/fnbeh.2023.1290548

**Published:** 2023-10-18

**Authors:** Marsida Kallupi, Elio Acquas, Liana Fattore

**Affiliations:** ^1^Department of Psychiatry, University of California, San Diego, La Jolla, CA, United States; ^2^Department of Life and Environmental Sciences, University of Cagliari, Monserrato, Italy; ^3^Institute of Neuroscience, National Research Council of Italy, Cagliari, Italy

**Keywords:** motivation, reward, decision making, goal-oriented behavior, mesolimbic dopamine system, brain activation and connectivity, impulsivity, behavior & cognition

The rapidly evolving field of Behavioral Neuroscience has made significant strides in understanding the complex mechanisms that govern human behavior, decision-making, and wellbeing. One area of particular interest is the study of Motivation and Reward, which has the potential to provide valuable insights into the underlying processes driving these behaviors. This Research Topic aims to highlight the latest advancements, challenges, and future perspectives in this area of study by showcasing a diverse range of original research and review articles submitted by esteemed researchers.

The manuscripts collected here encompass various aspects of Motivation and Reward, demonstrating the interdisciplinary nature of this field. Topics explored in the three original research articles include the application of machine learning techniques to understand the impact of stress hormones on animal behavior, the study of the differences between males and females in the long-term learning deficit induced by the co-exposure to the main consumed drugs during adolescence (i.e., alcohol, nicotine, and cannabinoids), and the role of race and ethnicity in shaping risk-taking behaviors in adolescents. Specifically, a first study by Bessa et al. used Siamese Fighting Fish (*Betta splendens*) as a biological model to study hormonal fluctuations during foraging. They found that an optimal level of cortisol maximizes foraging performance, while extreme levels of basal cortisol, both low and high, are associated with reduced average reward (i.e., leading to lower foraging performance). Importantly, to model foraging decisions, the authors applied the ε-greedy algorithm (Sutton and Barto, 2018) on a fish species whose brain structure and rich behavioral repertoire are comparable to those of mammals (Gerlai, 2014), offering many possibilities for research on vertebrate evolution, including cognitive and behavioral comparative analyses. Another animal species, rats, were used in the study of Abela et al., which suggests that smoking and having a few drinks and a joint at the weekend, which is common and considered not particularly harmful by adolescents/young adults, may have long-term and gender-dependent effects on the learning and reward systems. Finally, the Youth Risk Behavior Surveillance System (YRBSS) 2013 data was used by Aslam et al. to conduct a Latent Class Analysis (LCA) for the entire sample of teenagers, as well as separately for each sex. In this original research article, the authors identified five subsets of youth engaging in risky behavior: those who do not engage in risky behavior (i.e., Class 1 and 2—low risk), those with depressive symptoms and suicidal thoughts (Class 3), those with a high likelihood of using cigarettes and liquor (Class 4), and those who are polysubstance users (Class 5). Gender variations were also detected in the higher risk of mood disorders and depression among females.

The current state of neuroimaging research in the context of motivation and reward, reviewed by Weinstein, enriches the Research Topic of a comprehensive analysis of the literature on the fMRI studies conducted on healthy subjects, both on motivation and reward in tasks such as the *Sequential choice task* (Symmonds et al., 2010) or a *Monetary incentive delay task* (Mori et al., 2019), and on memory and attention in tasks such as a *Monetary and verbal reward task* (Albrecht et al., 2014) and a *Reaction-time task with goal-irrelevant expectancy violations in states of high- or low-reward motivation* (Murty and Adcock, 2014). The emerging complexity of contribution of the endocannabinoidome (Cristino et al., 2020) to the mesocorticolimbic dopaminergic system functioning, with critical implications on healthy behaviors as well as on neurological and psychiatric disorders, is elegantly reviewed by Kibret et al., which illustrates an up-to-date scenario of this contribution. The role of motivation and reward in promoting healthy behaviors was timely addressed by Michaelsen and Esch, who provide an insightful critical reading on the throughput that goal-oriented, stimulus-driven, and approach motivation play in nudging (Hansen and Jespersen, 2013), facilitating (Michie et al., 2005; Cane et al., 2012), and boosting (Grüne-Yanoff and Hertwig, 2016; Michaelsen and Esch, 2022) healthy changes in behavior.

Collectively, the potpourri of original research and reviews included in this Research Topic provides a comprehensive overview of the current state of Motivation and Reward research. By highlighting the latest discoveries, developments, and future challenges, this Research Topic seeks to inspire further research, promote funding opportunities, and guide researchers in their pursuit of unraveling the complex mechanisms that drive motivation and reward. Furthermore, this Research Topic emphasizes the importance of interdisciplinary collaboration, incorporating perspectives from biosciences, life sciences, medicine, psychiatry, and the mind to facilitate a more comprehensive understanding of motivation and reward mechanisms. By fostering an environment of curiosity and open-mindedness, we hope to encourage the development of innovative approaches and solutions to the challenges faced in this rapidly advancing field.

We believe that this Research Topic will serve as a valuable resource for researchers, academics, and professionals interested in the study of motivation and reward, providing direction and guidance for future investigations, and promoting the growth of knowledge in this essential area of Behavioral Neuroscience.

## Author contributions

MK: Writing—original draft, Writing—review and editing. EA: Writing—original draft, Writing—review and editing. LF: Writing—original draft, Writing—review and editing.
